# 30.3 ASSOCIATION BETWEEN SERUM C-REACTIVE PROTEIN, POSITIVE AND NEGATIVE SYMPTOMS OF PSYCHOSIS IN A GENERAL POPULATION-BASED BIRTH COHORT

**DOI:** 10.1093/schbul/sby014.124

**Published:** 2018-04-01

**Authors:** Golam Khandaker, Jan Stochl, Stanley Zammit, Robert Dantzer, Glyn Lewis, Peter B Jones

**Affiliations:** 1 University of Cambridge; 2 Cardiff University; 3 University of Houston in Texas MD Anderson Cancer Centre; 4 University College London

## Abstract

**Background:**

An association between low-grade inflammation and symptoms commonly shared between psychiatric disorders may explain the trans-diagnostic effects of inflammation, and lead to novel mechanistic hypotheses. Schizophrenia includes diverse symptoms, but the relationship between low-grade inflammation and specific psychotic symptoms has not been examined.

**Methods:**

In the general population-based ALSPAC birth cohort, serum CRP levels were assessed around age 16 years. Ten positive and ten negative symptoms of psychosis were assessed using self-report questionnaires around age 17 years. Associations between CRP and psychotic symptoms were examined using regression analysis before and after controlling for concurrent depressive symptoms, substance use, and other confounders. In addition, we used factor analysis to create positive and negative symptom dimension scores, which were then correlated with CRP.

**Results:**

About 13% of 5126 participants reported at least one positive symptom. Paranoid ideation (4.8%), visual (4.3%) and auditory hallucinations (3.5%) were the most common. Negative symptoms were correlated with concurrent depressive (P<0.001), and positive symptoms (P<0.001). CRP was associated with auditory hallucinations and anhedonia after controlling for potential confounders including concurrent depressive symptoms. The adjusted OR for auditory hallucinations for those with high compared with low CRP was 2.67 (95% CI, 1.27–5.60). Evidence for this association remained after excluding participants reporting positive symptoms in the context of cannabis/drug use, physical Illness or sleep, and after excluding participants reporting positive symptoms previously at age 12 years. The adjusted OR for anhedonia per SD increase in CRP was 1.13 (95% CI, 1.01–1.26). Factor analysis revealed similar findings. CRP was associated with both positive and negative symptom dimension scores. There was evidence for interaction between CRP and sex; the associations between CRP and psychotic symptoms were stronger in women.

**Discussion:**

Low-grade inflammation may be relevant for auditory hallucinations and anhedonia particularly in women. These findings need replication in other samples especially in patients with psychosis.

